# Multiple Extramedullary Plasmacytoma in a Setting of Complete Bone Marrow Remission: Food for Thought 

**Published:** 2017-10-01

**Authors:** Negi Preety, Kingsley Pamela Alice, Jomi Chinnu, Mathew Amrith, George Sneha, Immanuel Vivek, Abraham Puliyelil Abraham

**Affiliations:** 1 Department of Radiation Oncology, Christian Medical College & Hospital, Ludhiana, Punjab, India; 2 Department of Clinical Haematology and Haemato-Oncology and Bone Marrow Transplantation, Christian Medical College & Hospital, Ludhiana, Punjab, India

**Keywords:** Haematopoietic stem cell transplantation, Multiple myeloma, Extramedullary plasmacytoma, Positron emission tomography

## Abstract

Extramedullary plasmacytoma as a mode of relapse in multiple myeloma (MM) is unusual. Current recommendations do not incorporate the routine use of 18-fluorodeoxyglucose positron emission tomography-computed tomography (FDG PET/CT) imaging prior to haematopoietic stem cell transplant (HSCT) in MM. We report a case of relapsed MM with complete remission as per IMWG criteria. In the interim period, before the HSCT, the patient had localizing neurological signs and symptoms attributed to multiple extramedullary plasmacytomas. The uniqueness of this case is that this patient after complete marrow remission with no obvious external masses had unexpected, symptomatic multiple extramedullary plasmacytomas. This case illustrates the need for integration of FDG PET/CT imaging into routine pre-HSCT investigations in relapsed MM to prevent missing any asymptomatic extramedullary plasmacytomas.

## Case report

We describe the case of a 56-year-old female who complained of swelling over the right side of forehead for 15 days. Magnetic resonance imaging (MRI) brain showed lytic lesion involving left frontal bone with erosion of inner and outer tables suggestive of plasmacytoma. Laboratory work-up revealed an elevated β2-microglobulin level of 3018 ng/ml, raised kappa-lambda ratio- 29.640 and presence of multiple myeloma band. Bone marrow examination confirmed the diagnosis of plasma cell neoplasm with 42% plasma cells. Under the suspicion of MM, the diagnosis was proved histopathologically by specimen from tumour attached to the dura that revealed hypercellular marrow with increased plasma cells. Diagnosis of MM, stage III (International Staging System) was made. Further work-up with FDG PET/CT imaging demonstrated multiple hypermetabolic lytic osseous lesions suggestive of mitotic osseous involvement. 

Six cycles of Inj. Bortezomib 2 mg weekly along with Inj. Dexamethasone 40 mg weekly, and T. Thalidomide 100 mg (VTD regimen) were administered. She came to our hospital 1 year after the first treatment with relapse of MM. At this time, she was treated with 4 cycles of VTD regimen and achieved complete marrow remission. Further plan was to proceed with the HSCT. 

**Figure 1:**
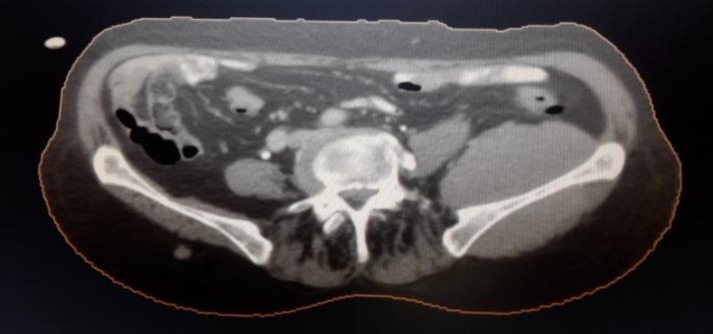
CT scan showing huge left paraspinal mass on the left side

**Figure 2:**
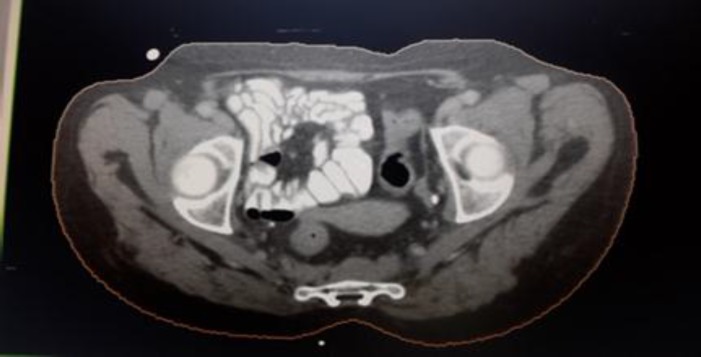
CT scan showing presacral mass

However, just before her planned transplant date, she developed weakness of both lower limbs, low backache which progressed to difficulty in walking over a couple of days. MRI Brain showed two lobulated extra-axial dural based soft tissue lesions in left frontal and parietal regions measuring 47 x 15 mm and 10 x 8 mm, respectively with perilesional edema. Whole spine screening showed diffuse altered signal intensity of all visualized vertebrae with ill-defined focal lesions and a soft- tissue component at the S3 – S5 level in the presacral region, extending into the left S3 and S4 neural foramina. There was similar soft tissue component in the left paraspinal region on left side at S1 level and in right paravertebral region at D7 vertebral level, measuring about 25 x 14 mm. There was thin enhancing epidural soft-tissue mass (approx. 5.7 mm) at the L5 - S1 level causing mild spinal canal compression with possible minimal extension along the neural foramina (Figures 1 & 2). In addition, her physical examination revealed a 2 x 2 cm deposit in the left hypochondrium. This was histologically confirmed as plasmacytoma (extramedullary relapse of MM). There was no evidence of clonal plasma cells in the marrow or monoclonal (M) serum protein. Palliative radiation therapy was applied to dural-based lesions in the left fronto-parietal region, presacral lesion and left pelvis by 3-dimensional conformal radiation therapy (3DCRT) to a dose level of 4500 cGy in 25 fractions. 

## Discussion

 Extramedullary plasmacytoma is involvement of any tissue outside the skeleton, with no clinical evidence of MM^10^. The diagnostic criteria for extramedullary plasmacytoma include plasma cell infiltration, normal skeletal survey, normal bone marrow (plasma cell infiltration < 5% of all nucleated cells), no related organ or tissue impairment and no M-protein in serum and / or urine^1^. Extramedullary relapse of MM is quite uncommon, comprising only 3% of all patients with plasma cell myeloma^11^. 

In our case, the patient was a 56-year- old MM woman with extramedullary plasmacytomas, involving the abdominal wall and pelvic region. This malignancy has been reported to be more common in male patients (75%) with a median age of 55 years. Of all extramedullary plasmacytomas, up to 80% have been recorded as arising in the head and neck region (nose, sinuses, and nasopharynx). Other sites reported are gastrointestinal tract, lungs, breasts, testes, and skin^2^^,^^12^. 

She received six cycles of VTD chemotherapy and achieved complete remission, which lasted approximately 12 months when she relapsed. Relapse is still a frequent problem for patients with MM, and whenever it occurs, there is almost nil chance for a cure with available current treatment options. However, remissions can be achieved in relapsed patients, but these tend to be shorter than first treatment responses^13^^,^^14^. Similar to this, we found a case of pulmonary plasmacytoma in a 66-year old man, who had been treated with VAD (vincristine, adriamycin, dexamethasone) chemotherapy for MM. This patient was declared free of disease after 4 cycles of chemotherapy. Three months later, the patient had multiple masses in the lungs visible on imaging^15^.

Extramedullary plasmacytoma during relapse of MM versus primary extramedullary plasmacytoma are considered two entirely different clinical entities due to relatively benign nature of primary extramedullary plasmacytoma as compared to extramedullary plasmacytoma as part of relapse of MM^16^. Even different types of extramedullary relapse of MM have significant differences in prognosis of these patients. Pour L et al. reported that patients with soft tissue-related extramedullary relapse had significantly poorer overall survival as compared to bone-related extramedullary relapse patients (30 vs. 45 months; p = 0.022) ^17^. In addition, overall survival from diagnosis was 5 months for soft tissue-related extramedullary relapse when compared to 12-month overall survival for bone-related extramedullary relapse. We can assume that early detection with accurate diagnosis of extramedullary plasmacytoma in a diagnosed patient of MM is of paramount importance since this finding usually carries a worse prognosis^18^. 

Nuclear imaging enables the identification of active disease by directly targeting the specific cellular properties of malignant plasma cells. FDG PET/CT imaging offers superior detection of myeloma bone disease and extramedullary manifestations^18^. Extramedullary plasmacytomas being diagnosed with FDG PET/CT imaging have been reported at sites such as gall bladder^19^. Detection of new disease activity in relapsed MM with the help of whole body skeletal survey, MRI or bone marrow biopsy is quite difficult. De Waal et al. reported that FDG PET-CT imaging might be helpful in such situation^20^. 

Currently, there is sufficient convincing data that FDG PET/CT imaging is emerging as an additional tool for detecting bone, bone marrow and extramedullary involvement in relapsed MM patients. There is ongoing research concerning the recommendations for the FDG PET/CT imaging prior to HSCT since a high incidence of extramedullary relapses have been reported following autologous and allogeneic stem cell transplant ^21^^,^^22^.

Search of literature revealed a case report of a 43- year-old MM patient who had early relapse after autologous stem cell transplantation. At the time of relapse, he was planned for allogeneic stem cell transplantation. The patient was asymptomatic with negative examination findings. FDG PET/CT imaging was performed as a part of standard work-up prior to allogeneic stem cell transplantation which detected symmetrical FDG uptake in the testes^23^. As in our case report, these authors reported the value of FDG PET/CT in face of pre-allogenic stem cell transplantation thereby allowing the treating clinicians to detect an early and asymptomatic extramedullary relapse. 

Our case illustrates two main learning points: Firstly, the incidence of extramedullary plasmacytoma in a setting of relapsed MM is rare and is even more so in a relapsed and treated MM with complete marrow remission. Secondly, before embarking on an expensive treatment modality with the aim of cure such as HSCT implementation of comprehensive imaging like FDG PET/CT can serve as a one-stop shopping imaging technique to rule out any extramedullary plasmacytoma. 

Based on the findings of complete bone marrow remission, if our patient had proceeded to undergo HSCT it would have resulted in failure with extramedullary disease clinically manifesting after the expensive marrow directed treatment. This case shows that FDG PET/CT imaging is an appropriate tool for detecting asymptomatic extramedullary plasmacytoma in a known patient of MM following complete remission in bone marrow before embarking on HSCT. 

## CONCLUSION

 Experience regarding the role of FDG PET/CT imaging in patients with extramedullary plasmacytoma at relapse of MM is scanty. This case report highlights the importance of FDG PET/CT imaging prior to embarking on bone marrow haematopoietic stem cell transplantation in patients with relapsed multiple myeloma and complete remission in bone marrow to rule out any asymptomatic extramedullary disease. 
